# The Monthly Stethoscope and Medical Reporter

**Published:** 1856-04

**Authors:** 


### "THE MONTHLY STETHESCOPE & MEDICAL REPORTER."

We acknowledge the reception of this new candidate for public favor. The editors are already known as the conductors of the late Stethescope and Virginia Medical Gazette. From what we know of the reputation of the two gentlemen, as well as from the appearance and contents of the number received, we have no hesitation in saying that they will furnish a journal of high order, and we cordially extend to them the right hand of fellowship and commend their enterprise to the patronage of the profession. It is published in Richmond, Virginia, monthly, and contains 64 pages, and is fuınished at $3 per annum, in advance. Goorich A. Wilson, M. D. and Richard A. Lewis, M. D., Editors.

We should be glad to receive the first number, and a continuance of exchange.

